# A Peptide-Based Hydrogel for Adsorption of Dyes and Pharmaceuticals in Water Remediation

**DOI:** 10.3390/gels8100672

**Published:** 2022-10-19

**Authors:** Anna Fortunato, Miriam Mba

**Affiliations:** Dipartimento di Scienze Chimiche, Università degli Studi di Padova, Via Marzolo 1, 35131 Padova, Italy

**Keywords:** peptide, hydrogel, supramolecular gel, water remediation, dye adsorption, pharmaceuticals adsorption, diclofenac, methylene blue

## Abstract

The removal of dyes and pharmaceuticals from water has become a major issue in recent years due to the shortage of freshwater resources. The adsorption of these pollutants through nontoxic, easy-to-make, and environmentally friendly adsorbents has become a popular topic. In this work, a tetrapeptide–pyrene conjugate was rationally designed to form hydrogels under controlled acidic conditions. The hydrogels were thoroughly characterized, and their performance in the adsorption of various dyes and pharmaceuticals from water was investigated. The supramolecular hydrogel efficiently adsorbed methylene blue (MB) and diclofenac (DCF) from water. The effect of concentration in the adsorption efficiency was studied, and results indicated that while the adsorption of MB is governed by the availability of adsorption sites, in the case of DCF, concentration is the driving force of the process. In the case of MB, the nature of the dye–hydrogel interactions and the mechanism of the adsorption process were investigated through UV–Vis absorption spectroscopy. The studies proved how this dye is first adsorbed as a monomer, probably through electrostatic interactions; successively, at increasing concentrations as the electrostatic adsorption sites are depleted, dimerization on the hydrogel surface occurs.

## 1. Introduction

Pharmaceutically active compounds (PhACs) are emerging contaminants that can be found in industrial and domestic wastewater, surface water, ground water, and, more problematically, in drinking water [1]. They include nonsteroidal anti-inflammatory drugs (NSAIDs) (ibuprofen, diclofenac, naproxen), antibiotics (amoxicillin, ciprofloxacin, erythromycin, nalidixic acid), anticonvulsants (carbamazepine, primidone), antidepressants (diazepam, meprobamate), and hormones (estrogen), among others. Tonnes of pharmaceutically active compounds are used every year to treat human and animal diseases in hospitals, households, aquaculture, or feedstock facilities. However, they are not fully adsorbed, and the unmetabolized drugs are excreted, entering wastewater or surface and groundwater. Inappropriate disposal of unwanted drugs is another source of contamination [2]. These wastewaters end up in wastewater treatment plants (WWTPs), which are not able to efficiently remove this class of pollutants before final recirculation as drinking water. For example, a concentration of 67 μg/L of carbamazepine was found in the effluents of a WWTP in Madrid, which treats water in an area where there is a university campus, several pharmaceutical industries, and a hospital [3]. Furthermore, leaching or inappropriate waste management in the pharmaceutical industry also contributes to the introduction of PhACs into the environment. For example, a concentration of 17.48 mg/L of ciprofloxacin was detected in wastewater effluents from a pharmaceutical industry in Croatia [4]. Low concentrations of PhACs in drinking water do not constitute an immediate public health risk; however, the effects due to long-term exposure are not known. On the other hand, the adverse effects of PhACs in the environment have already been reported in the literature, including the development of antibiotic resistance [5]. Thus, the development of processes for the removal of PhACs has become of major importance. New processes are needed to remove low concentrations of PhACs (ng to μg per liter) arriving to WWTPs, but there is also a need for in situ facilities that are capable of removing higher concentrations of these pollutants in industry effluents (μg-mg per liter) [6].

In addition to PhACs, dyes are a critical source of water pollution that make water unfit for drinking. They are widely used in different industries, particularly the textile industry. Due to their toxicity, the presence of organic dyes in water causes health disorders and disrupts aquatic life [7]. Among these dyes, methylene blue (MB) is released in quantity by industry into water sources, posing a health threat due to its toxicity, non-biodegradability, and carcinogenic property [8].

Various advanced technologies have been developed for the removal of dyes and PhACs from water, including advanced oxidation techniques, membrane filtration, adsorption, and membranes with sorption capacities [9,10,11]. Adsorption stands out due to its low energy consumption, easy operation, and high efficiency [12,13]. In this context, hydrogels have emerged as a suitable alternative. Hydrogels are ideal adsorbents because of their high hydrophilicity, large surface area, and the presence of multiple functional groups on the porous structure [14,15,16].

Short peptide self-assembly is a rapidly extending research field. Short peptides combine inherent biocompatibility and biodegradability with ease of synthesis and scalability. In addition, peptide self-assembly is well-understood, and both properties and self-assembly can be tuned through control of the peptide sequence [17,18,19,20,21,22]. Short peptides self-assemble in water, through a combination of different noncovalent interactions, to give hydrogels [23,24,25]. These supramolecular soft materials have mainly found applications in biomedical fields [26,27,28,29], but also find applications in the development of conductive materials [30,31], catalysis [32], crystal growth [33], and water remediation. The efficiency of peptides and peptide-based hydrogels for oil spill recovery [34,35,36], removal of dyes [37,38,39], and metal cations [38,40] from water is well-documented in the literature. However, their application to the removal of PhACs from water is still at its beginning. Only recently, Giuri et al. reported the use of Boc-protected tripeptide hydrogels for the removal of diclofenac from water [41].

**Figure 1:**
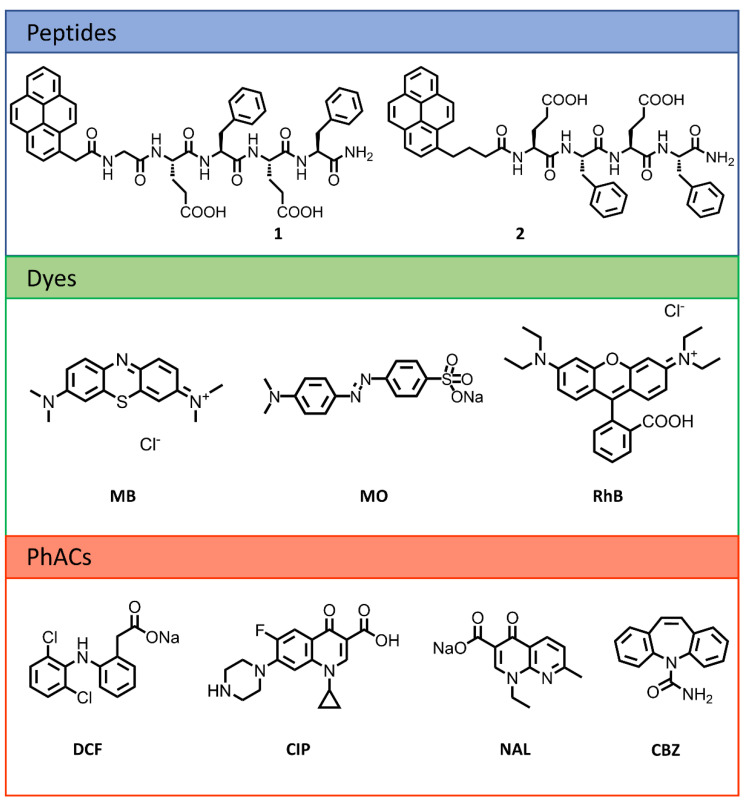
Structure of previously reported peptide **1**, and structures of peptide **2**, dyes, and PhACs employed in the present work.

We recently reported the pyrene–pentapeptide conjugate **1** (Figure 1), and demonstrated that metal cations trigger its self-assembly in water to form metallo-hydrogels that efficiently adsorbed cationic dyes from water [42]. Although hydrogel formation was also achieved by simple pH variation in the absence of cationic metals, these pH-triggered hydrogels suffered from low mechanical stability and fell apart upon dye adsorption. To increase the environmental compatibility of the adsorbent, it is desirable to develop a metal-free peptide hydrogel. In this paper, we report a novel pyrene–tetrapeptide conjugate **2** able to form self-supporting hydrogels at acidic pH. The hydrogels efficiently removed cationic and zwitterionic dyes from water, and promising results were obtained for the adsorption of PhACs (Figure 1).

## 2. Results and Discussion

### 2.1. Peptide Design and Synthesis

The structure of peptide **2** has alternating polar charged glutamic acids and hydrophobic phenylalanine residues. This pattern is known to favor the formation of β-sheets [43]. Here, the C-terminal has been amidated, while the N-terminal has been capped with a pyrene moiety, so the solubility of **2** in water is governed by the charge state of the Glu side chains. The large aromatic surface of pyrene can establish additional π–π interactions that facilitate self-assembly and interaction with aromatic pollutants. In addition, the fluorescence of this chromophore may provide information about self-assembly and allows for easy detection of peptide leaching [44]. The main differences between molecules **1** and **2** are the absence of the Gly residue in **2** and the introduction of a larger methylene spacer between the chromophore and the peptide. These structural modifications favor hydrogel formation at a lower gelator concentration of **2** compared to **1**.

Peptide **2** was synthesized using standard Fmoc-based solid-phase peptide synthesis protocols on rink-amide resin, as described in the Materials and Methods section. The peptide was obtained in 85% yield, and its identity was assessed by nuclear magnetic resonance spectroscopy (NMR), electrospray mass spectrometry (ESI-MS), and Fourier transform infrared spectroscopy (FT-IR) (see Scheme S1 and Figures S2–S4 of the Supporting Information).

### 2.2. Gelation Ability

The presence of two hydrophilic pH-sensitive amino acid residues (Glu) provides good solubility in basic water and introduces pH responsiveness.

**Figure 2:**
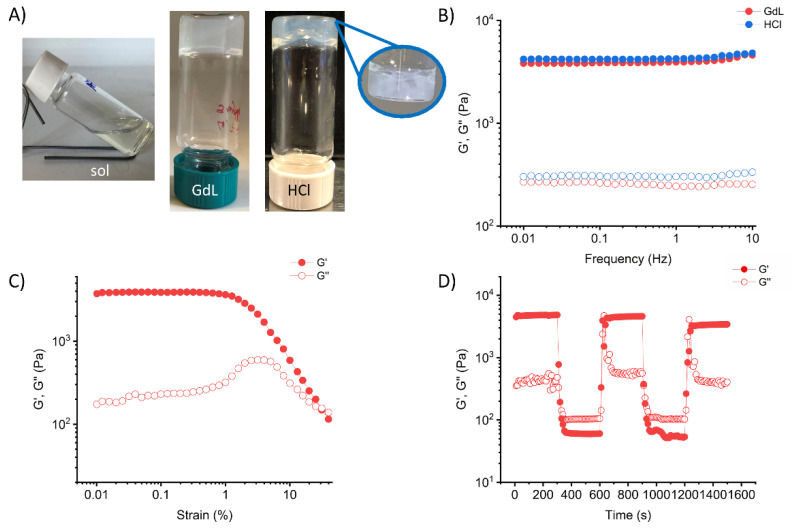
(**A**) Photographs of vials containing from left to right: the peptide at 0.5% in solution, the GdL gel, and the HCl gel; (**B**) frequency sweep of the HCl gel (blue) and the GdL gel (red) at 0.5% concentration, where G’ values are indicated with filled circles and G’’ is indicated with open circles; (**C**) strain sweep of the GdL gel at 0.5% concentration; (**D**) dynamic strain amplitude cyclic test of the GdL gel at 0.5% concentration.

As expected, **2** is not soluble in Milli-Q water alone (even after heating), but a transparent solution was obtained after the addition of 1.8 equivalents of NaOH 1M solution in combination with sonication. Acidification of the solution using both HCl and glucono-δ-lactone (GdL) led to the formation of self-supporting hydrogels, as assessed by the vial inversion test (Figure 2A and Figures S5 and S6 of the Supporting Information). HCl gels were formed in 10 min, while GdL gels required up to 18 h. Visually, the HCl gel was non-homogeneous, whereas the GdL gel was semitransparent and uniform. This phenomenon is well-known in the literature. When a strong acid such as HCl is used to trigger gelation, gel formation takes place faster than the diffusion of the acid in the solution, and gel spots can be found where the acid was added. Instead, GdL dissolves and diffuses into the solution before hydrolysis, leading to homogeneous gels. The minimum gelation concentration was found to be 0.1% for the GdL gel and 0.3% for the HCl gel. Interestingly, a gel–sol transition was not observed when heating any of the gels (Tgel); instead, at temperatures above 70 °C, the gels shrank, expelling part of the entrapped water (see Figure S5 of the Supporting Information).

### 2.3. Gel Characterization

The following studies were performed at a concentration of 0.5%, as the gels could be manipulated without damage.

The gel nature of the samples was confirmed by oscillatory rheology. Frequency sweep experiments (Figure 2B) showed that the elastic modulus (G’) of both gels was almost independent of the frequency in the studied range, and approximately one order of magnitude higher than the viscous modulus (G”), as expected for supramolecular gels [45]. Notably, the behavior of the two samples in amplitude sweep experiments differed significantly. The GdL gel displayed a linear viscoelastic behavior up to 1% of strain. Along with increasing applied strain, both G’ and G’’ deviated progressively from linearity until the crossover point was reached at 30% of the strain, where the gel–sol transition took place (Figure 2C). In contrast, we were unable to obtain amplitude sweep tests for the HCl sample, since hydrogel fracture occured even at low strain values. Again, the differences between the two samples may be related to the different gelation kinetics. The formation of macroscopic gel domains in the case of HCl gels also resulted in the formation of regions that were weaker and easily broken under strain. We also evaluated the self-recovery ability of the GdL gel using dynamic strain amplitude experiments (Figure 2D). The gel was subjected to several cycles of high strain values (150% strain) to force the gel–sol transition, which were alternated with low strain values (0.1% strain) to allow recovery. As shown in Figure 2D the GdL gel maintained its stability and showed mechanical recovery, though the storage modulus decreased by 8% after three cycles.

**Figure 3:**
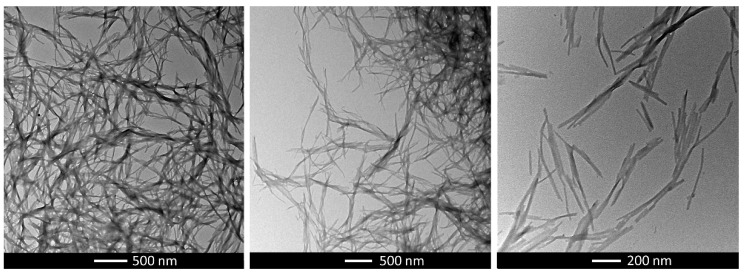
TEM micrographs of GdL xerogels at 0.5% concentration.

Because of the superior mechanical properties and reproducibility of gelation of GdL gels, we decided to continue our studies using exclusively these samples (Data for the HCl gels are given in Figures S7–S9 of the Supporting Information). From the morphological point of view, the GdL gel was investigated via transmission electron microscopy (TEM). Micrographs of the xerogels showed the formation of dense networks of tape-like fibrils (Figure 3). A closer look reveals narrower flat structures sprouting from the tape-like fibrils. Because this peptide is expected to give β-sheet bilayer structures, with the hydrophobic residues buried inside, a possible packing mode is the formation of β-sheet bilayers that are associated through edge-to-edge interactions, mediated by pyrene moieties [46].

**Figure 4:**
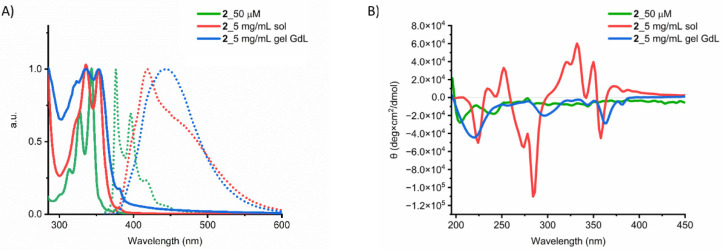
(**A**) UV–Vis absorption (solid line), emission spectra (dotted line), and (**B**) CD spectra of peptide **2** in solution and the GdL gel.

The π–π interactions were studied using UV–Vis absorption and emission spectroscopies (Figure 4A and Figure S10 of the supporting Inormation). First, we investigated the behavior of a 50 μM solution of peptide at basic pH. The solution displays the typical absorption profile of pyrene: the S0→S2 transition gives an intense band between 360 nm and 290 nm with the 0-0 peak at 343 nm, whereas the peaks at 327 nm, 313 nm, and 300 nm are the vibronic replicas [44]. At a higher concentration (0.5%), the UV–Vis of the basic solution is characterized by a broadening and redshift of the absorption band. The 0-0 peak moves to 352 nm, and the relative intensity between 0-0 and its vibronic replicas changes. Concerning the emission spectra, the diluted solution shows a band ranging from 350 nm to 500 nm, which corresponds to the nonaggregated pyrene monomer. On the other hand, the 0.5% solution exhibits a broad and unstructured profile with a peak located at 420 nm and a long tail up to 650 nm. For the gelled sample, the broadening of the absorption band further increases, while in the emission spectra, a new broad band ranging from 350 to 700 nm appears. On the basis of these results, we can support the hypothesis that peptide monomers exist only in diluted basic solutions, while at higher concentrations, the hydrophobic pyrene cores tend to interact to each other through π–π stacking, an interaction that is reinforced upon gelation.

The secondary structure was studied by CD spectroscopy (Figure 4B). In the monomer state, the CD spectrum shows the typical profile of a random coil with disordered structure, characterized by a minimum at 204 nm. When the concentration of the solution was increased to 0.5%, a minimum at 225 nm was observed, indicating the formation of β-sheet structures [47,48]. However, the spectrum is dominated by strong Cotton effects observed in the absorption region of the pyrene chromophore, which are one order of magnitude more intense than the band at 225 nm. Thus, in agreement with absorption and emission data, CD suggests that in concentrated solution, strong π–π interactions between pyrene moieties become established, resulting in a supramolecular chiral arrangement of the chromophores [49]. Upon gelation, the negative band moves to shorter wavelengths, suggesting an increase of β-sheet content. Cotton effects are still observed in the pyrene absorption region, but with an intensity comparable to that of the β-sheet band.

### 2.4. Adsorption of Dyes

**Figure 5:**
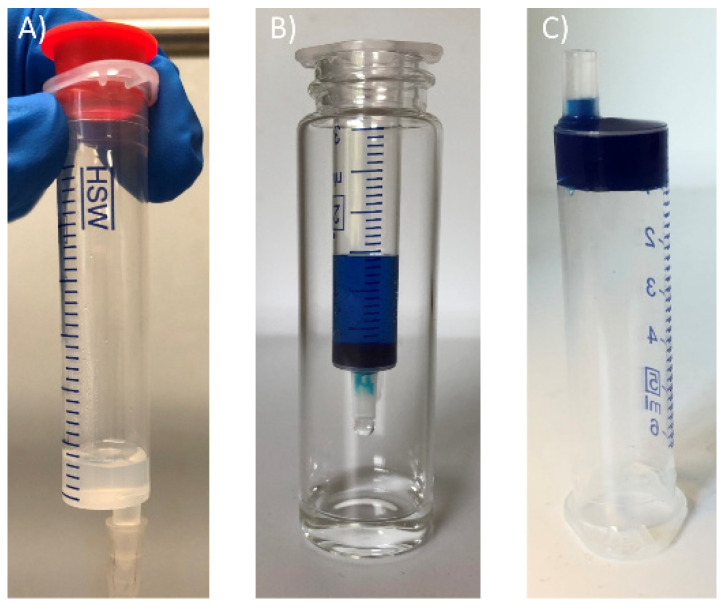
Adsorption experiment setup: (**A**) the gel is placed inside a syringe; (**B**) the solution of MB is allowed to flow through the gel; (**C**) upon elution, the gel maintains its mechanical stability and does not flow when the syringe is turned upside down.

First, GdL gels were tested for the adsorption of methylene blue (MB). Three different experiment setups were investigated: (a) the gel was placed inside a syringe, and the MB solution was allowed to flow through the gel (Figure 5); (b) a preformed gel was immersed in a beaker containing the MB solution; (c) the gel was formed in a vial and the MB solution was casted on top of it. These preliminary studies were carried out under the same conditions (1 mL of GdL gel, 5 mL of MB solution at 50 mg/mL). A simple comparison by the naked eye of the solutions of pollutants after 24 h confirmed that, between the three setups, the first gave the best performances. Therefore, it was adopted for the subsequent studies (see Supporting Information for more details).

Next, we extended our studies to methyl orange (MO) and rhodamine B (RhB) and determined the dye removal efficiency (RE) by UV–Vis absorption spectroscopy using Equation (1) as follows:

(1) $$RE\;\left(\%\right)=\frac{\left(C_{0}-C_{f}\right)}{C_{0}}\times 100$$

where C_0_ is the initial concentration of pollutant and C_f_ is the concentration of pollutant in the eluted solution. A very good efficiency was obtained for the adsorption of MB (RE of 90%), while for both MO and RhB, the gel showed a poorer performance. The RE was 50% for RhB and only 16% for the negatively charged MO (Table 1, entries 1–3). No leaching of the gel components was detected in any case and the gel maintained its mechanical stability after dye adsorption, as assessed by inversion of the syringe (Figure 5C and Figure S11 of the Supporting Information). The better adsorption of MB can be justified by electrostatic interactions between the positively charged MB and deprotonated sites present on the GdL gel, which act simultaneously to hydrogen bonding, while the acidic groups present on RhB and MO may prevent efficient adsorption because of electrostatic repulsions between negative charges.

In the case of MO, UV–Vis analysis revealed not only a decreased concentration of the pollutant but also a red-shift of the absorption maxima. Analysis of the pH showed that the solution of the pollutant is more acidic upon contact with the hydrogel (pH 4.5). We attributed the shift of the absorption band to the acidic conditions, and, in agreement, the UV–Vis spectrum of MO registered at pH 4.5 was superimposable to that registered upon elution through the gel (see Supporting Information for details). At this pH, MO (pKa = 3.47) is not fully protonated, but both the neutral and anionic forms coexist.

The effect of the initial concentration of the dye on adsorption was investigated for MB. Data are summarized in Table 1, entries 3–5. To investigate the effect of concentration, additional solutions at 25 mg/L and 100 mg/L were prepared, and 5 mL were eluted through 1 mL of gel. Data reveal that RE decreases when increasing the initial concentration of MB from 25 to 50 mg/L, and then increases slightly when going from 50 to 100 mg/L.

**Figure 6:**
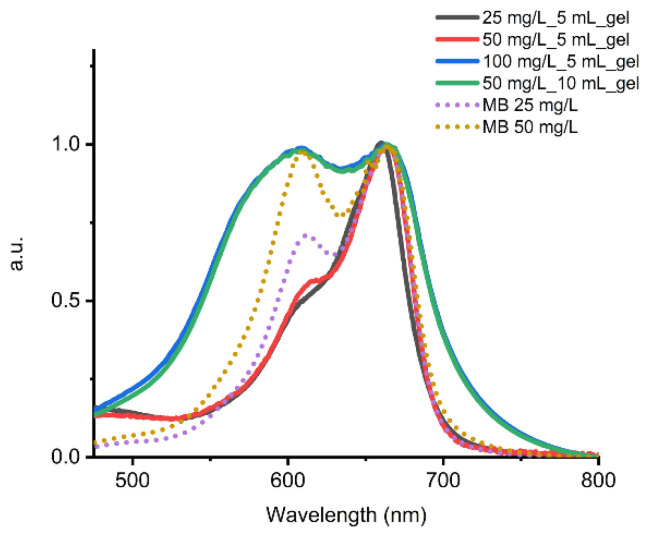
UV–Vis absorption spectra of the initial MB solutions (dotted line) and MB adsorbed on the hydrogel (solid line) upon elution of 5 mL of a 25 mg/L (black), 50 mg/L (red), and 100 mg/L (blue) solution of MB, and upon elution of 10 mL of a 50 mg/L (green) solution of MB.

The decrease of RE as initial concentration of MB increases can be related to the saturation of active adsorption sites on the hydrogel. The increase of RE with initial concentration deserves more attention. It is noted in the literature that depending on the concentration, MB exits in aqueous solution as monomer, dimer, trimer, or tetramer [50]. Moreover, it may adsorb to the adsorbent surface in any of its forms [51,52,53]. To gain more insight, we registered the UV–Vis spectra of the adsorbed dye and compared them to the UV–Vis spectra of the initial solutions of MB. Figure 6 shows the UV–Vis spectra of MB in aqueous solution at 25 and 50 mg/L (the one at 100 mg/L gave a saturated signal) and adsorbed MB on the hydrogel after elution of 5 mL of initial solutions of different concentrations.

In the initial solutions (Figure 6, dotted lines), MB shows two bands at 664 nm and 610 nm that have been attributed to the monomer and dimer forms, respectively [50]. As initial concentration decreases, the intensity of the monomer band increases at the expense of the dimer band, indicating, as expected, a decrease in dimer content as MB concentration decreases. In the UV–Vis absorption spectrum of the hydrogel upon elution of a 25 mg/L solution, the dimer band is attenuated significantly, and the spectrum resembles those reported for the monomeric MB in aqueous solution or adsorbed on a surface [54]. Thus, MB initially adsorbs as a monomer even if the initial solution has a high content of dimer. We hypothesized that this first adsorption process is governed by strong MB-adsorbent electrostatic interactions that predominate over the MB–MB interaction responsible for the dimer formation. Upon elution of a 50 mg/L solution, the hydrogel shows that the dimer band intensity increases slightly. When dye concentration is further increased to 100 mg/L, the spectrum changes significantly. The absorption band is broader; monomer and dimer bands have similar intensities, and an intense blue-shifted tail appears. Thus, at higher concentrations, MB first saturates electrostatic adsorption sites, and only the monomer form is found on the surface of the hydrogel. Afterwards, probably with the high concentration as driving force, extra MB molecules are adsorbed, now through π–π interactions and formation of the dimeric form of MB on the surface of the adsorbent is favored. The blue-shifted tail may indicate the formation of higher order aggregates of MB, but we cannot rule out MB–pyrene interactions.

We also investigated how the variation of the volume of initial solution affects the adsorption capability of the hydrogel (Table 1, entries 3, 6, 7). To do so, 2.5, 5, and 10 mL of a MB solution at 50 mg/L were eluted through 1 mL of GdL–gel. We found the same trend when initial MB concentrations were varied at a fixed volume. Thus, the adsorption process seems to be mainly affected by the mass ratio of adsorbent/MB, and it takes place as described in the previous paragraph. Indeed, the UV–Vis spectrum of the hydrogel upon elution of 10 mL of a 50 mg/L solution of MB is superimposable to that obtained after elution of 5 mL of a 100 mg/L solution of the dye (Figure 6).

### 2.5. Adsorption of PhACs

We then tested the GdL gel for the adsorption of a series of representative PhACs. We chose the NSAIDs diclofenac sodium salt (DCF), the antibiotics ciprofloxacin (CIP) and nalidixic acid sodium salt (NAL) and the anticonvulsant carbamazepine (CBZ). In all cases, 5 mL of a 50 mg/mL pollutant solution was eluted through 1 mL of GdL gel, and the pollutant concentration in the eluted solution was determined by UV–Vis spectroscopy. Under these conditions, we found that the best performances were obtained with DCF (RE 75%), followed by CIP (RE 40%), while CBZ and NAL showed the worst results (RE 21% and 24%, respectively) (Table 2, entries 1–4). That CBZ, with the largest π-surface, is adsorbed worse than DCF suggests that π–π interactions are not the main forces involved in the adsorption process. On the other hand, electrostatic interactions may be somewhat favorable for the case of CIP, containing a basic amine, but this do not justify the better performance in the adsorption of DCF. Both DCF and CIP structures contain groups capable of stablish hydrogen bonds, but also contain halogen atoms, so halogen bonding may also play an important role in the adsorption process. To study the possible role of halogen bonding, we tested the adsorption of the dehalogenated DCF analogue. However, we found that this compound is not stable under the working conditions, and the results were not conclusive (see Supporting Information for details).

The effect of the initial concentration on adsorption was investigated for DCF, which gave the best RE among the PhACs tested. Data are summarized in Table 2, entries 4–6. The data reveal that RE increases as the initial concentration increases. On the other hand, when the volume of the solution was changed at a fixed concentration of 50 mg/L, the RE increased when the volume decreased (Table 2, entries 4, 7, 8).

The increase in RE with initial pollutant concentration has been explained in the literature as an improved diffusion and mass transfer process that is favored at higher concentrations, accompanied by an augmented number of collisions between pollutant molecules and the adsorbent, which favors adsorption [55,56]. When the volume of the solution is increased at a fixed initial concentration, we hypothesized that the concentration of the solution on top of the gel does not remain constant throughout the experiment and that diffusion of DCF into the hydrogel may occur faster than the elution process, such that the concentration of DCF in the remaining solution decreases overtime. As a consequence, the driving force of the concentration loses its positive effect.

## 3. Conclusions

We have described a new peptide–pyrene hybrid gelator that forms stable hydrogels at acidic pH. Hydrogels obtained by acidification with GdL were successfully employed for the removal of a number of dyes and PhACs from water. In particular, the best results were achieved for the dye MB and the anti-inflammatory drug DCF. The effect of initial concentration and volume was studied for the two aforementioned molecules. Data revealed that adsorption of MB is mainly affected by the mass ratio of adsorbent/hydrogel. First, the monomeric form of the cationic dye adsorbed through electrostatic interactions until saturation of the charged adsorption sites. Afterwards, higher concentrations of dye were adsorbed through dimerization /polymerization of the dye on the hydrogel surface. On the other hand, DCF adsorption was favored at higher concentrations, and no saturation behavior was detected in the range of concentrations investigated. In the case of DCF, the concentration appears to be the driving force of the adsorption process: it improves the mass transfer and increases the chance of collision between DCF molecules and the active sites of the hydrogel.

## 4. Materials and Methods

### 4.1. General Methods

ESI-MS spectra were recorded using an ESI-TOF MarinerTM BiospectrometryTM Workstation of Applied Biosystems by flow injection analysis (mobile phase methanol with 0.1% formic).

^1^H and ^13^C were recorded in deuterated dimethyl sulfoxide at 25 °C at a frequency of 300 MHz. The residual solvent peak was used as internal reference. Chemical shifts (δ) are expressed in parts per million (ppm). The multiplicity of a signal is indicated as s (singlet), d (doublet), t (triplet), dd (doublet of doublets), dt (doublet of triplets), td (Triplet of doublets), q (quartet) and m (multiplet). The acronym “br” indicates a broadened signal.

FT-IR spectra were measured on a FT-IR Perkin-Elmer 1720X spectrophotometer, in KBr disk, using a resolution of 2 cm^−1^, a total of 100 scans were averaged.

UV–Vis absorption spectra were measured on a Varian Cary 50 spectrophotometer at 25 °C. For solution samples, a reduced volume quartz cell with 0.1 cm, 0.4 cm, or 1 cm optical path was used. Gels were analysed using a rectangular cell with detachable windows and 0.2 mm of optical. Gels were prepared in a glass vial; a small amount was transferred to the sample chamber and the cell was carefully closed to avoid the formation of bubbles

Emission spectra were measured on a Varian CaryEclipse spectrophotometer at 25 °C. A quartz cell with optical path of 10 × 2 mm and volume 500 μL or 10 × 4 mm and volume 1400 μL was used for the solutions. Gels were analysed using a 500 μL quartz cell with an optical path of 10 × 2 mm. Gels were prepared directly inside the cuvette

CD spectra were measured on a Jasco J-1500 instrument at 25 °C and were baseline corrected, a total of 64 measurements were averaged. The spectra are expressed in terms of total molar ellipticity (deg⸱cm^2^⸱dmol^−1^). Solutions were analysed using a reduced volume quartz cell with 0.1 cm, 0.4 cm or 1 cm optical path. Gels were analysed using a rectangular cell with detachable windows and optical path of 0.2 mm. Gels were prepared in a glass vial; a small amount was transferred to the sample chamber, and the cell was carefully closed.

TEM images were recorded on a Jeol 300 PX instrument using glow discharged carbon coated grids. Gels were diluted prior to analysis; 10 μL of the sample were then deposited directly on a grid, and the excess of sample was removed with #50 hardened Whatman filter paper; no staining was used. The images were analyzed with the ImageJ program.

Oscillatory rheology was performed on a Kinexus Lab+ rheometer with parallel plate geometry. 1 mL of hydrogel was prepared in a glass mold with a diameter of 2 cm and placed onto the lower plate. A thermal cover was used, and temperature was set at 25 °C using a Peltier temperature controller. Frequency sweeps were recorded between 10–0.001 Hz at a constant strain. Strain sweeps were carried out between 0.01–110% at a constant frequency of 1 Hz. Step strain experiments were performed applying cycles of deformation and recovery steps. The first step (rest conditions) was performed at a fixed frequency of 0.1 Hz and at a strain of γ = 0.1% (within the LVE region) for a period of 5 min. The deformation step was performed at a fixed frequency of 0.1 Hz, applying a constant strain of γ = 150%, (above the LVE region) for a period of 5 min. Deformation and recovery steps were repeated three times.

### 4.2. Synthesis of ***2***

All reagents and solvents were purchased from Irish Biotech or Merck and were used as received. Compound **2** was synthesized by solid phase 9-fluorenylmethoxycarbonyl (Fmoc) chemistry on Rink amide resin using stablished procedures [42]. 

ESI-MS: [M + Na]^+^ calculated for C_48_H_49_N_5_O_9_ 862.34, found: 862.5.

^1^H-NMR (DMSO-*d*_6_, 300 MHz): δ 12.01 (bs, COOH), 8.36 (d, 1H, *J* = 9.3 Hz), 8.31–7.88 (m, 11H), 7.33 (d, 1H, *J* = 8.1 Hz), 7.29–6.94 (m, 12H, overlapping signal Ar of Phe and NH_2_), 4.63–4.33 (m, 2H, H_α_, Phe), 4.33–4.10 (m, 4H, H_α_ Glu), 3.06–2.89 (m, 2H, H_β_, Phe), 2.87–2.65 (m, 2H, H_β_, Phe), 2.35–2.07 (m, 6H, overlapping signal H_γ_ Glu and methylene linker), 2.04–1.92 (m, 2H, methylene linker), 1.89–1.5 (m, 4H, H_β_, Glu) ppm.

^13^C-NMR (DMSO-*d*_6_, 75 MHz): δ 174.43, 174.40, 173.08, 172.76, 171.83, 171.46, 171.12, 138.18, 138.13, 137.04, 131.35, 130.90, 129.76, 129.62, 129.56, 128.63, 128.47, 128.38, 128.04, 127.92, 127.67, 126.96, 126.67, 126.59, 125.39, 125.23, 124.69, 124.62, 124.01, 54.12, 52.62, 52.43, 37.97, 37.53, 35.22, 32.66, 30.61, 30.51, 27.91, 27.77, 27.58, 18.31, 12.53.

FT-IR (KBr): ν^~^ (cm^−1^) = 3395, 3286, 3086, 3061, 3032, 2940, 1734, 1719, 1650, 1617, 1544, 1498, 1453, 1443, 1415, 1340, 1322, 1315, 1273, 1244, 1236, 1207, 1201, 1182, 1170, 842, 742, 719, 699.

### 4.3. Gel Preparation

HCl-triggered gelation: A known amount of **2** was introduced in a 4 mL glass vial. Then, 900 μL of MilliQ water was added, followed by NaOH 1M (1.8 eq). A clear solution was obtained with the aid of sonication. Then, MilliQ water and HCl solution (0.5M, 2 eq.) were added to obtain a self-supporting gel with the desired final concentration. Gel formation was assessed by the vial inversion test.

GdL-triggered gelation: A known amount of **2** was introduced in a 4 mL glass vial. Then, 900 μL of milliQ water was added. NaOH 1M (1.8 eq.) was added, and a clear solution was obtained with the aid of sonication. The desired final concentration was reached through the addition of milliQ water. Then, a known amount of GdL (3 eq.) was added, and the mixture was vortexed and left at room temperature overnight. Gel formation was assessed by the vial inversion test.

### 4.4. Gel Preparation in Syringes

A volume of 1 mL gel at 0.5% concentration was prepared as follows: 5 mg of **2** was introduced to a 4 mL vial. Then, 900 μL of MilliQ water was added. NaOH 1M (1.8 equiv. 11 μL) was added, and a clear solution was obtained with the aid of sonication. MilliQ water was then added to reach a volume of 1 mL. Then, GdL (3 eq., 3.5 mg) was added, and the mixture was vortexed and immediately transferred into a 5 mL syringe that was sealed at the bottom. The mixture was allowed to rest at room temperature overnight to allow gel formation. Gel formation was assessed by the syringe inversion test.

### 4.5. Adsorption Experiments

Dye and PhAC stock solutions at 200 mg/L were prepared in MilliQ water. All other solutions were prepared by dilution of stock solutions. For all pollutants, UV–Vis absorption calibration curves were obtained in the range of concentrations between 5 mg/L and 50 mg/L. By plotting the maximum absorbance vs. concentration, the molar extinction coefficient for each dye and PhAC was determined (see Supporting Information for details). Adsorption experiments were performed in triplicate; only the average RE value is given. Here, 1 mL of gel at 0.5% concentration was prepared in a syringe. A known volume of dye or PhAC solution was loaded on top of the gel, and the solution was allowed to flow through the gel by gravity. The eluted solution was recovered in a vial. When the pollutant solution had passed through the gel, the eluted solution was analyzed by UV–Vis spectroscopy. The concentration of the pollutant was determined using the Lambert Beer equation A = εCl, where A is the absorbance, ε the molar extinction coefficient (mol L^−1^ cm^−1^), C the concentration of pollutant (mol L^−1^), and l the path length (cm).

## Figures and Tables

**Table 1:** Removal efficiency of dyes. 1 mL of GdL-gel at 0.5% (adsorbent dosage 5 mg) was used.

Entry	Dye	C_0_ (mg/L)	V (mL)	m_dye_ (μg)	RE ^1^ (%)
1	MO	50	5	250	16
2	RhB	50	5	250	50
3	MB	50	5	250	90
4	MB	25	5	125	100
5	MB	100	5	500	91
6	MB	50	2.5	125	99.5
7	MB	50	10	500	90

^1^ Average values over 3 tests.

**Table 2:** Removal efficiency of PhACs. 1 mL of GdL gel at 0.5% (adsorbent dosage 5 mg) was used.

Entry	Dye	C_0_ (mg/L)	V (mL)	m_PhAC_ (μg)	RE ^1^ (%)
1	NAL	50	5	250	21
2	CBZ	50	5	250	24
3	CIP	50	5	250	40
4	DCF	50	5	250	75
5	DCF	25	5	125	59
6	DCF	100	5	500	89
7	DCF	50	2.5	125	83
8	DCF	50	10	500	53

^1^ Average values over 3 tests.

## Data Availability

The data presented in this study are available in the article.

## Supplementary Materials

The following are available online at https://www.mdpi.com/article/10.3390/gels8100672/s1, Scheme S1: synthesis of **2**, Figure S1: ESI-MS of compound **2**, Figure S2: ^1^H NMR (DMSO-d6, 300 MHz) of **2**, Figure S3: ^13^C-NMR (DMSO-d_6_, 300 MHz) of **2**, Figure S4: FT-IR spectrum of **2** (KBr disk), Figure S5: Photographs of 0.1% GdL gel before and after heating Figure S6: Photos of HCl-triggered gel, Figure S7: Normalized absorption (solid-line) and emission (dotted-line) spectra of HCl-triggered gel (0.5%), Figure S8: CD spectrum of HCl-triggered gel (0.5%), Figure S9: TEM images of HCl-triggered gel, Figure S10: Non-normalized emission spectra of **2** diluted solution (green), 0.5% solution (red) and GdL gel 0.5% (blue), Figure S11: (Top) inversion of the syringe after the process to demonstrate the stability of the gel after the adsorption of pollutants; (bottom) UV-vis spectrum of eluted MB showing the absence of **2** in the eluted sample, Table S1: Calibration curve slope and relative wavelength, Table S2. Removal efficiency and adsorption capacities for the adsorption of dyes and PhACs. Figure S12: UV-vis normalized absorption spectra of dyes and PhACs used, Figure S13: Schematic representation of gel formation, Scheme S2: synthesis of dehalogenated DCF, Figure S14: ESI-MS of dehalogenated DCF, Figure S15: ^1^H NMR (MeOD, 300 MHz) of dehalogenated DCF.
