# Supplementary material for: A Peptide-Based Hydrogel for Adsorption of Dyes and Pharmaceuticals in Water Remediation
Source: Gels. 2022 Oct 19;8(10):672. doi: 10.3390/gels8100672 (PMC9601570; doi:10.3390/gels8100672)
Supplement: Supplementary file 1 [file gels-08-00672-s001.zip › gels-1953914-supplementary.pdf]

Supporting informations

Contents

General methods **S3**

Synthetic Procedure and Characterization of 2 .... **S5**

Scheme S1: Synthesis of **2** **S6**

Figures S1: ESI-MS spectra of **2** S7

Figures S2: ^{1}H-NMR spectrum of **2** S8

Figures S3: ^{13}C-NMR spectrum of **2** S9

Figures S4: FT-IR spectra of **2**........ S10

Preparation and Characterization of Gels **S11**

Preparation of gels S11

Figures S5: Photographs of 0.1% GdL gel before and after heating........ S11

Figures S6: Photographs of HCl-triggered gel........ S12

Figures S7: Normalized absorption and emission spectra of HCl-triggered gel (0.5%)........ S12

Figures S8: CD spectrum of HCl-triggered gel (0.5%). S12

Figures S9: TEM images of HCl-triggered gel........ S13

Figures S10: Non-normalized emission spectra of **2** diluted solution, 0.5% solution and GdL gel 0.5% S13

Figures S11: Syringe after the process and UV-Vis spectrum of eluted MB........ S14

Table S1: Calibration curve slope and relative wavelength S15

Figures S12: UV-VIs normalized absorption spectra of Dyes and PhACs used S16

Synthetic Procedure and Characterization of dehalogenated DCF........ **S17**

Scheme S2: Synthesis of dehalogenated DCF........ S17

Figures S13: ESI-MS spectra of dehalogenated DCF........ S17

Figures S14: ^{1}H-NMR spectrum of dehalogenated DCF S18

GENERAL METHODS

- Mass Spectrometry

ESI-MS experiments were performed with a ESI-TOF MarinerTM BiospectrometryTM Workstation of Applied Biosystems by flow injection analysis using methanol with formic acid (1 %) as mobile phase.

- NMR Spectroscopy

^1H and ^{13}C NMR were recorded at 289 K on a Bruker Avance III 300 spectrometer using the partially deuterated solvent as internal reference. Deuterated DMSO has been used as solvent (99.9% d_6, Sigma Aldrich). Chemical shifts (δ) are expressed in parts per million (ppm). The multiplicity of a signal is indicated as: s (Singlet), d (Doublet), t (Triplet), dd (Doublet of doublets), dt (Doublet of triplets), td (Triplet of doublets), q (Quartet) and m (Multiplet). The acronym "br" indicates a broadened signal. The spectral width for ^1H-NMR is from 0ppm to 14ppm, whereas the ^{13}C-NMR spectral width is from 0 ppm to 210 ppm.

- FT-IR

FT-IR absorption spectrum were recorded with a FT-IR Perkin-Elmer, model 1720X spectrophotometer, in KBr disk, at a nominal resolution of 2 cm^{-1}, averaging 100 scans.

- UV-Vis Spectroscopy

UV-Vis absorption spectra were recorded with a Varian Cary 50 spectrophotometer at 25°C. All spectra are baseline corrected. A rectangular cell with detachable windows (Hellma) and optical path of 0.02 cm (Hellma) was used for the analysis of gelled samples. For non-gelled samples a reduce volume quartz cell with 1 cm, 0.5 cm or 0.1 cm optical path was used.
General methodology for gel samples: gels were prepared in a glass vial; a small amount was transferred to the sample chamber and the cell was closed with the top window taking care of not forming bubbles.

- Emission Spectroscopy

Emission spectra were recorded in a Varian CaryEclipse spectrophotometer at 25°C. A quartz cell with optical path of 10x2 mm and volume 500 µL was used for gel samples. A quartz cell with optical path of 10x10 mm and volume 3 mL was used for solutions.
General methodology for gel samples: Gels were prepared directly inside the cuvette without amendment such as dilution.

- Circular Dichroism Spectroscopy

CD spectra were recorded on a Jasco J-1500 instrument at 25°C and were baseline corrected. The spectra are expressed in terms of total molar ellipticity ($deg \cdot cm^2 \cdot dmol^{-1}$). For non-gelled samples a reduce volume quartz cell with 1 cm or 0.1 cm

optical path was used. A rectangular cell with detachable windows (Hellma) and optical path of 0.02 cm (Hellma) was used for the analysis of gelled samples.

General methodology for gel samples: gels were prepared in a glass vial; a small amount was transferred to the sample chamber and the cell was closed with the top window taking care of not forming bubbles.

- TEM

Transmission electron microscopy (TEM) images were recorded with a Jeol 300PX instrument. A glow discharged carbon coated grid was floated on a small drop of solution and excess was removed by using #50 hardened Whatman filter paper. Gels were diluted prior to analysis, a small amount of each sample has been deposited directly on a glow discharged carbon coated grid and no staining has been used. The excess has been removed by #50 hardened Whatman filter paper. The images obtained have be analysed with ImageJ program.

- Rheology

Rheological analyses were carried out on a Kinexus Lab+ rheometer with a parallel plate geometry. Hydrogel samples were prepared on a total volume of 1 mL and immediately transferred onto the plate. An anti-evaporation chamber was used to prevent drying of the samples and temperature was set at 25 °C. Frequency sweep tests were carried out between 10-0.001 Hz at a constant strain. Strain measurements were carried out between 0.01-110% at a constant frequency of 1 Hz. Step strain experiments were performed on hydrogels subjecting the sample to consecutive deformation and recovery steps. The first step (rest conditions) was performed at a constant strain γ=0.1% (within the LVE region) and at a fixed frequency of 0.1 Hz for a period of 300 s. The deformation step was performed applying a constant strain of γ=150%, (above the LVE region) for a period of 300 s at a fixed frequency of 0.1 Hz. Deformation and recovery steps were repeated three times.

SYNTHETIC PROCEDURE AND CHARACTERIZATION OF 2

Compound 2 was synthesized using standard solid phase 9-Fluorenylmethoxycarbonyl (Fmoc) chemistry on Rink amide resin. When not in use the resin was dried and stores in freezer with the amino-terminus Fmoc-protected. The MBHA Rink Amide Resin was purchased from Irish Biotech (commercial loading 0.68 mmol/g); O-benzotriazole-N,N,N',N'-tetramethyluronium hexafluorophosphate (HBTU), 1-Hydroxybennzotriazole hydrate (HOBt), N,N-diisopropylethylamine (DI-PEA), piperidine, trifluoroacetic acid (TFA), triisopropylsilane (TIS), Fmoc-amino acids and solvents were purchased from Sigma-Aldrich or Iris Biotech. SPPS was performed in a standard vessel for manual SPPS equipped with a glass frit and two outlets. The stirring was achieved by bubbling nitrogen from below, thus all the steps have been carried out under N2 flux. A washing step implies 1 min of stirring and then removal of solvent. In general, 10 mL of solvent must be used for 1 gram of resin. The resin was swelled dumping 1 g of resin into the SPPS vessel and then 10 mL of dimethylforma-mide (DMF) were added for resin swelling and stirred gently for 30 minutes. Fmoc deprotection was achieved by treatment of the resin with a mixture piperidine/DMF (2:8) for 20 minutes (x2), then washing with DMF (x3). For amino acid couplings we used the following protocol: 3.0 eq. (relative to the resin loading) of Fmoc-protected amino acid were activated in a separated vial with 2.9 eq. of HBTU, 2.9 eq. of HOBt and 9 eq. of DIPEA in DMF. This mixture was then added to a peptide reaction vessel containing the Rink amide resin and mixed for 2 hours. All coupling and deprotection steps were monitored using a Kaiser test on a few resin beads which were removed from the peptide chamber after drying with dichloromethane (DCM). If necessary, the coupling step was repeated. The coupling with pyrene functionalized core was per-formed using 2.5 equiv of 1-Pyrenebutyric acid, 2.4 equiv of HBTU and HOBt and 7.5 equiv of DIPEA. The reaction was performed for 1 hour. The solvent was removed and the resin was washed with DMF (3 x 10 mL), DCM (3 x 10 mL) and DMF (2 x 10 mL). Cleavage from the resin and removal of side-chain protecting groups was accom-plished by reaction with a cocktail of TFA/ water/ TIS (95:2.5:2.5) for 3 hours. The sol-vents were collected in a flask and the resin was washed with DCM (3 x 10 mL). Sol-vents collected were concentrated in rotavapor (a potassium hydroxide trap was used) to the half. DCM was added and the volatiles were evaporated again. The process was repeated 3 times, after which the solvents were evaporated to dryness. The product was precipitated from cold diethyl ether and the precipitated peptide was isolated by centrifugation and lyophilized. A white powder was obtained (85% yield).

ESI-MS: $[M+Na]^+$, calculated for $C_{48}H_{49}N5O_9$ 862.34, found: 862.5.

^{1}H-NMR (DMSO-d_6, 300 MHz): δ 12.01 (bs, COOH), 8.36 (d, 1H, J = 9.3 Hz), 8.31-7.88 (m, 11H), 7.33 (d, 1H, J = 8.1 Hz), 7.29-6.94 (m, 12H, overlapping signal Ar of Phe and NH2), 4.63-4.33 (m, 2H, Hα, Phe), 4.33-4.10 (m, 4H, Hα Glu), 3.06-2.89 (m, 2H, Hβ, Phe), 2.87-2.65 (m, 2H, Hβ, Phe), 2.35-2.07 (m, 6H, overlap-ping signal Hγ Glu and methylene linker), 2.04-1.92 (m, 2H, methylene linker), 1.89-1.5 (m, 4H, Hβ, Glu) ppm

^{13}C-NMR (DMSO-d_6, 75 MHz): δ 174.43, 174.40, 173.08, 172.76, 171.83, 171.46, 171.12, 138.18, 138.13, 137.04, 131.35, 130.90, 129.76, 129.62, 129.56, 128.63, 128.47, 128.38, 128.04, 127.92, 127.67, 126.96, 126.67, 126.59, 125.39, 125.23, 124.69, 124.62, 124.01, 54.12, 52.62, 52.43, 37.97, 37.53, 35.22, 32.66, 30.61, 30.51, 27.91, 27.77, 27.58, 18.31, 12.53.

FT-IR (KBr): $\tilde{\nu}$ (cm^{-1}) = 3395, 3286, 3086, 3061, 3032, 2940, 1734, 1719, 1650, 1617, 1544, 1498, 1453, 1443, 1415, 1340, 1322, 1315, 1273, 1244, 1236, 1207, 1201, 1182, 1170, 842, 742, 719, 699.

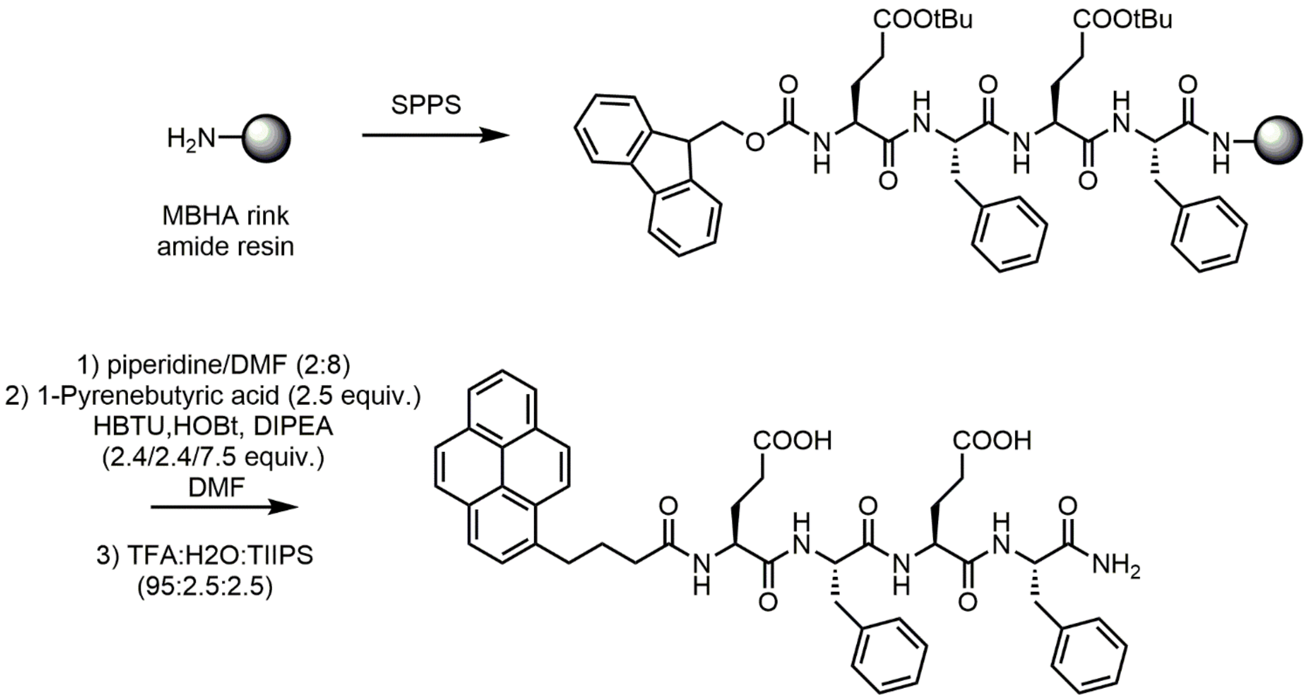

Scheme S1: synthesis of **2**.

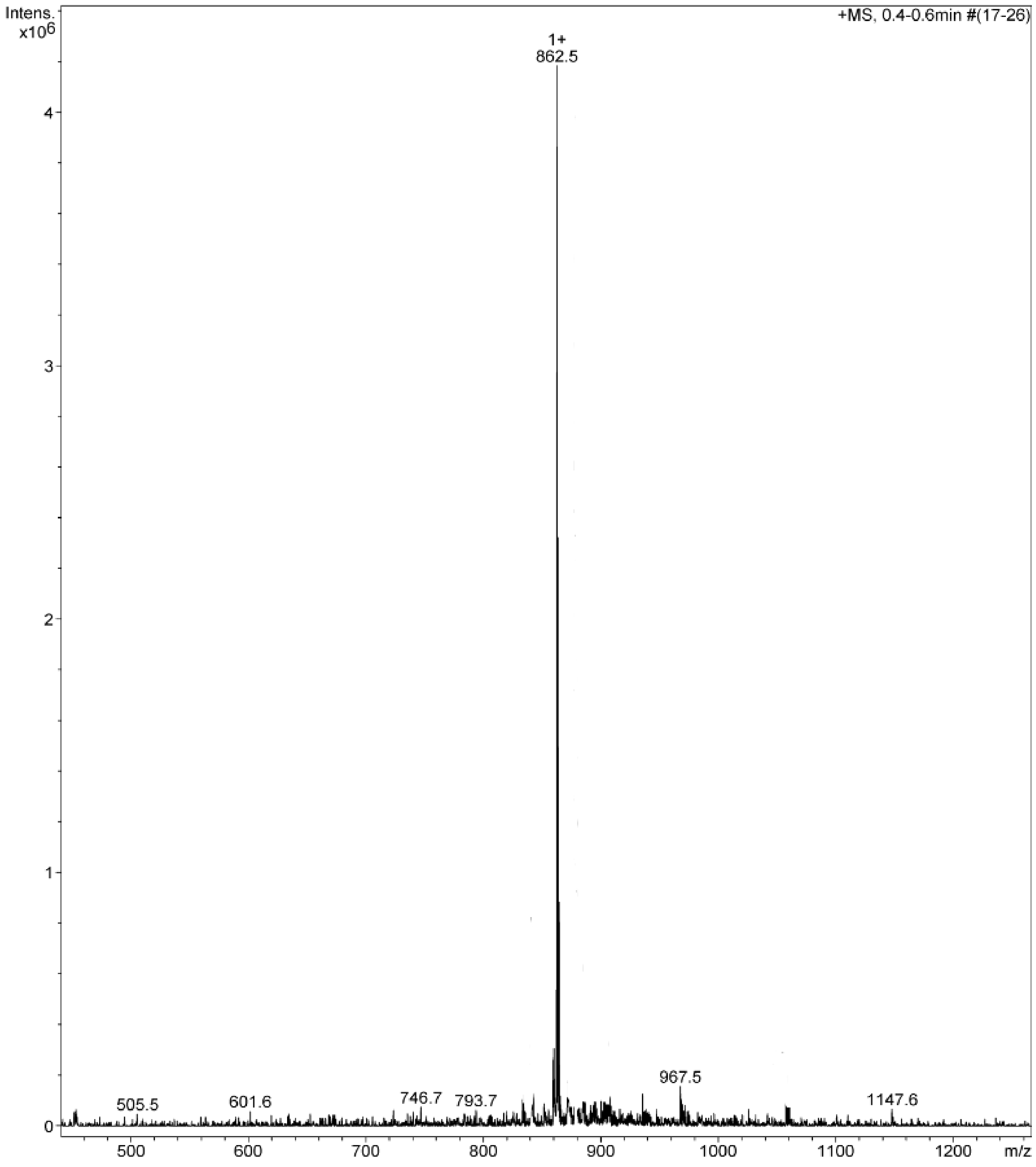

Figure S1: ESI-MS compound **2** in MeOH.

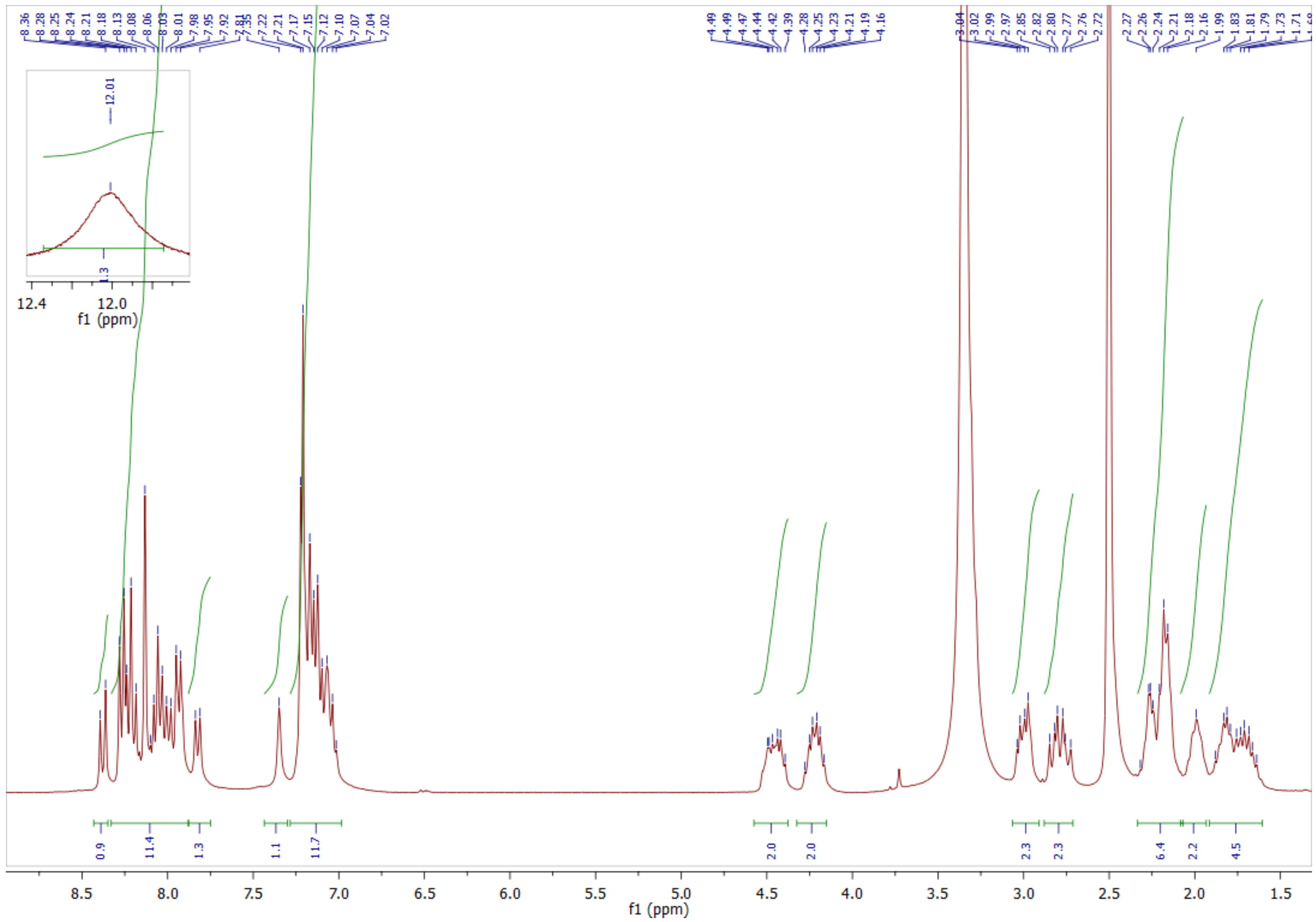

Figure S2: ^{1}H NMR (DMSO-d_6, 300 MHz) of **2**.

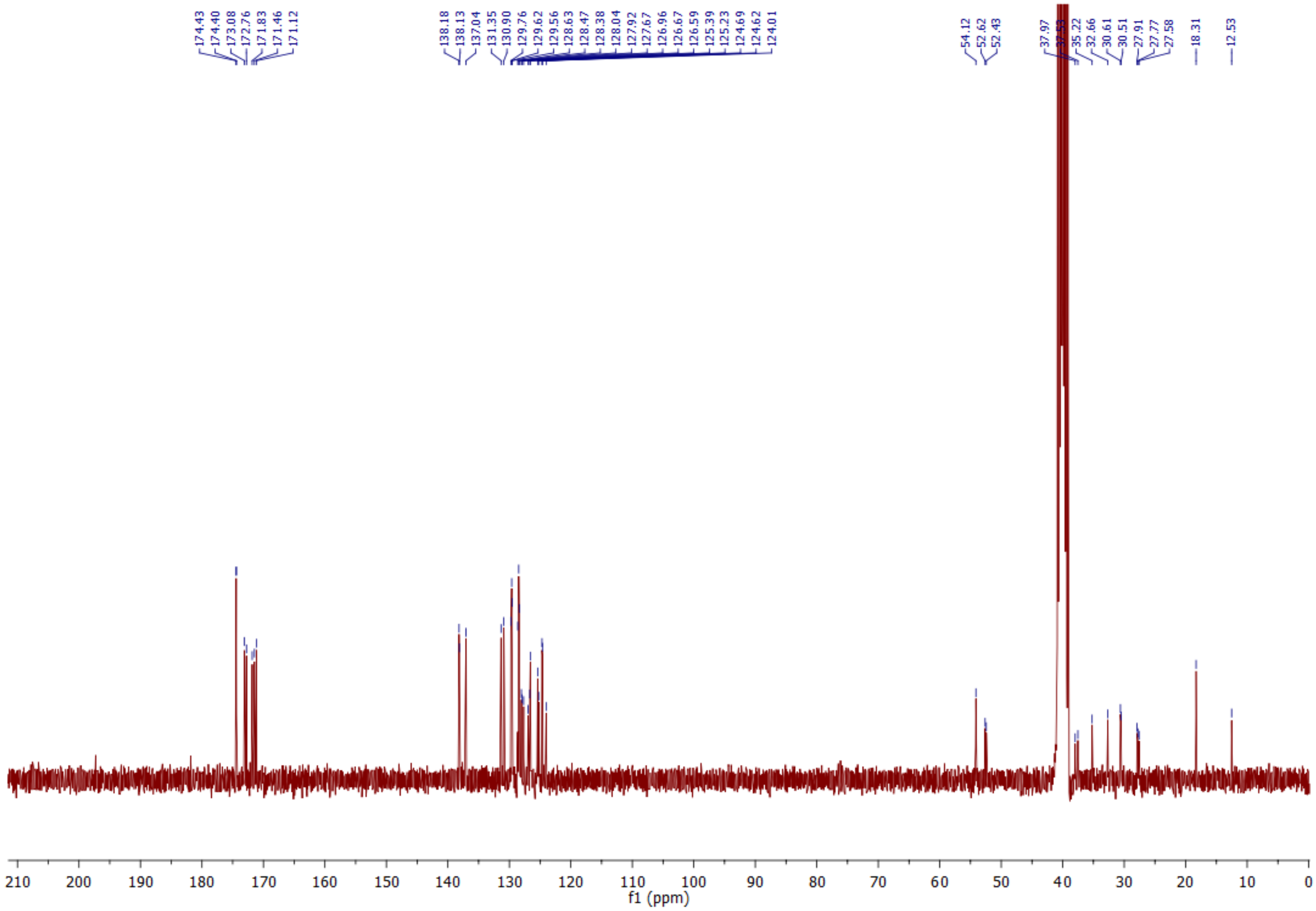

Figure S3: ^{13}C-NMR (DMSO-d_6, 75 MHz) of **2**.

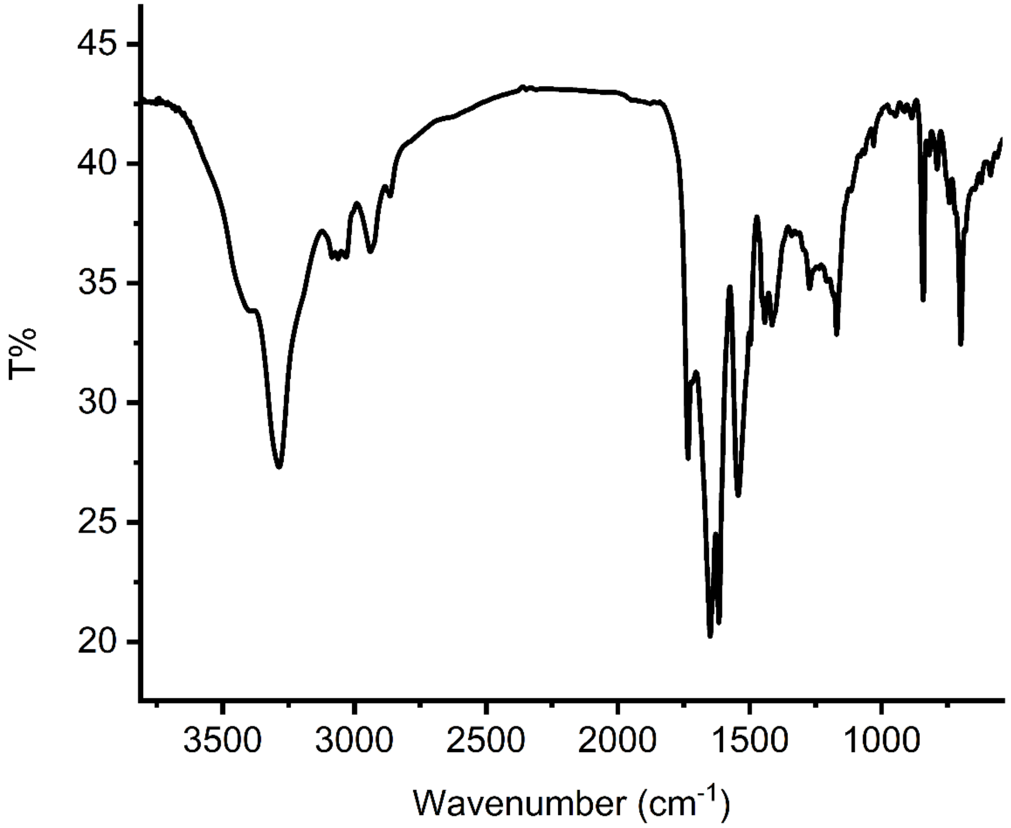

Figure S4: FT-IR spectrum of **2** (KBr disk).

PREPARATION AND CHARACTERIZATION OF GELS

HCl triggered gelation. A known amount of **2** was introduced in a 4 mL vial. Then 900 μL of milliQ water were added. NaOH 1M (1.8 eq.) was added to reach a clear solution through sonication. Then an amount of milliQ water and HCl solution (0.5M, 2eq.) were added to obtain a self-supporting gel with the desired final concentration. Gel formation was assessed by the vial inversion test.

GdL triggered gelation. A known amount of **2** was introduced in a 4 mL vial. Then 900 μL of milliQ water were added. NaOH 1M (1.8 eq.) was added to reach a clear solution through sonication. The desired final concentration was reached through the addition of milliQ water. Then, a known amount of GdL (3eq.) was added and its dissolution was favoured through vortex. Gel formation was assessed by the vial inversion test.

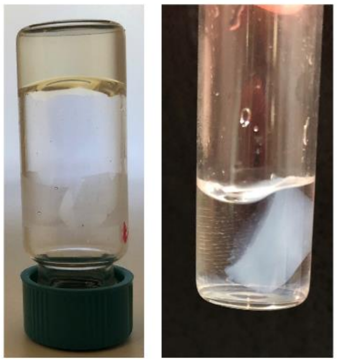

Figure S5: Photographs of 0.1% GdL gel before (left) and after heating (right). Gel shrinks expelling part of the entrapped water.

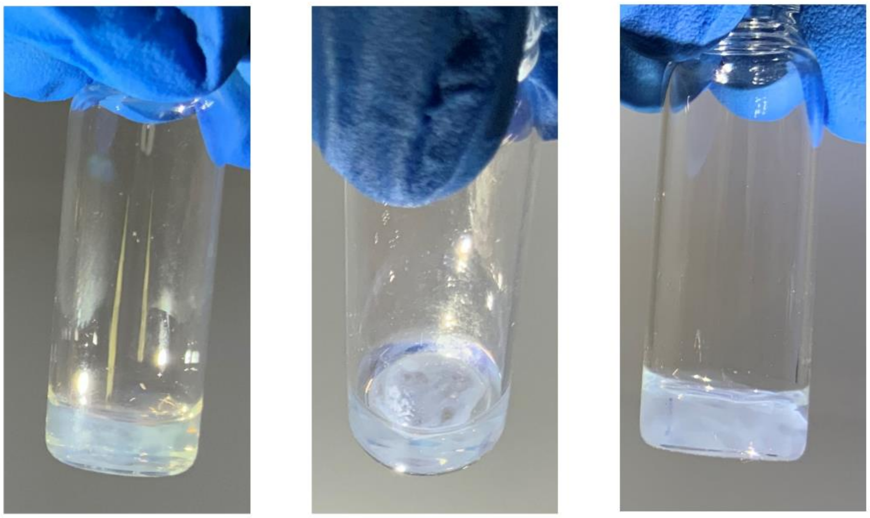

Figure S6: Photographs of inhomogeneous HCl-triggered gel.

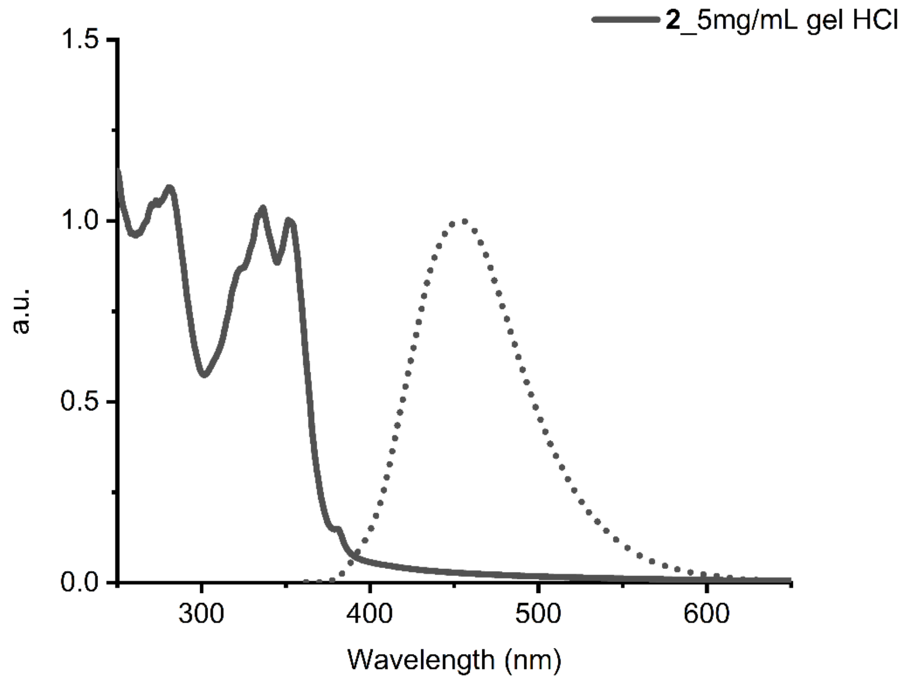

Figure S7: Normalized absorption (solid-line) and emission (dotted-line) spectra of HCl-triggered gel (0.5%).

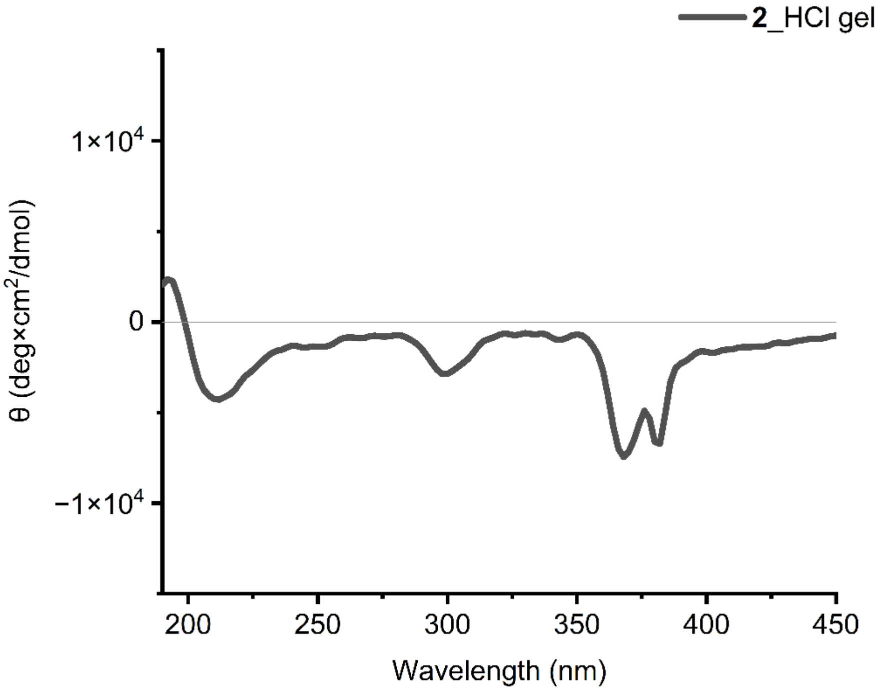

Figure S8: CD spectrum of HCl-triggered gel (0.5%).

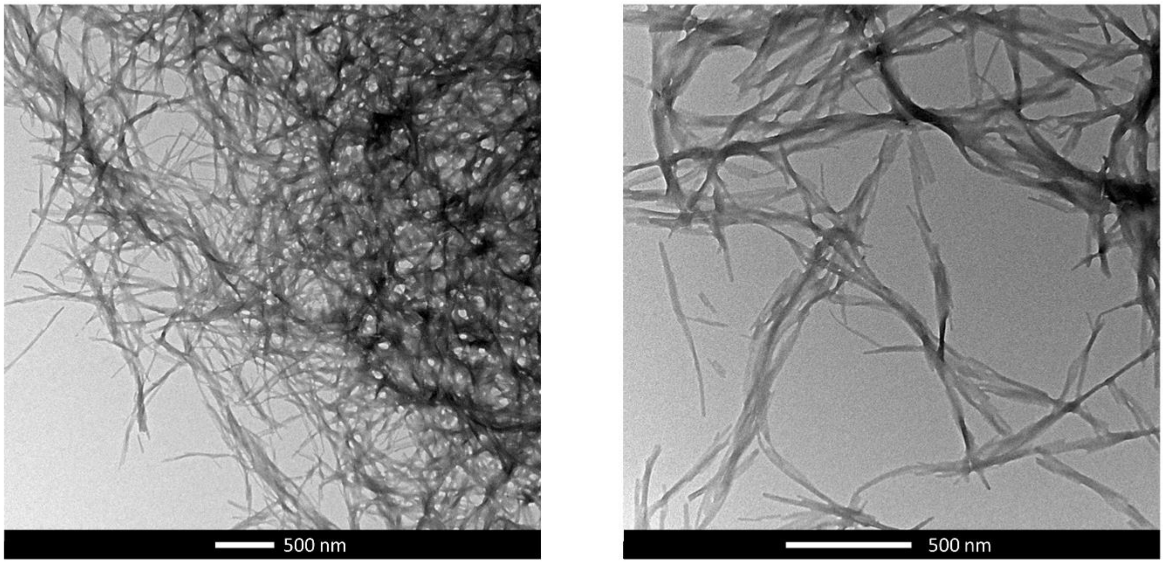

Figure S9: TEM images of HCl-triggered gel.

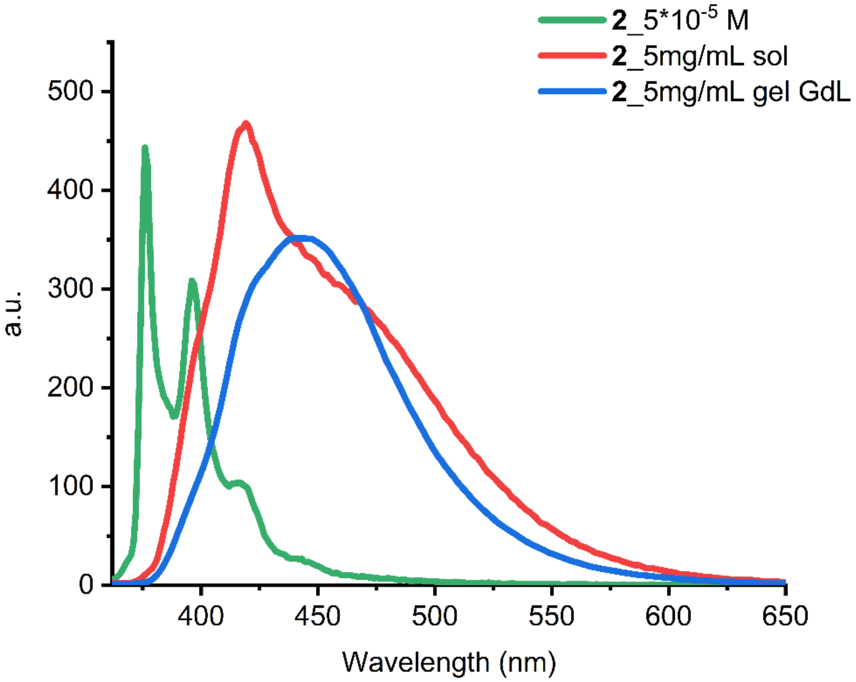

Figure S10: Non-normalized emission spectra of **2** diluted solution (green), 0.5% solution (red) and GdL gel 0.5% (blue).

ADSORPTION OF POLLUTANTS

Gel preparation in syringe for water remediation: 1 mL of a gel at 0.5% concentration was prepared as follows. 5 mg of 2 were in-troduced in a 4 mL vial. Then 900 µL of MilliQ water were added. NaOH 1M (1.8 equiv. 11 L) was added and a clear solution was obtained with the aid of sonication. MilliQ water was then added till 1 mL. Then GdL (3equiv., 3.5 mg) was added, the mixture was vortexed and immediately transferred into a 5mL syringe sealed at the bottom. The mixture was allowed to rest at room temperature overnight to allow the gel formation. Gel formation was assessed by the syringe inversion test. Dye and PhAC stock solutions at 200 mg /L were prepared in MilliQ water. All other solutions were prepared by dilution of stock solutions. For all pollutants, UV-Vis absorption calibration curves were obtained in the range of concentrations between 5 mg/L and 50 mg/L., plotting the maximum absorbance versus concentration the molar extinction coefficient for each dye and PhAC was determined (see Supporting Infor-mation for details). Adsorption experiments were performed in triplicate, and only the average RE value is given. 1 mL of gel at 0.5% concentration was prepared in a syringe. A known volume of dye or PhAC solution was loaded on top of the gel, and the solu-tion was allowed to flow through the gel by gravity. The eluted solution was recovered in a vial. When the pollutant solution had passed through the gel, the eluted solution was analysed by UV-Vis spectroscopy. The concentration of the pollutant was deter-mined using the Lambert Beer equation $A=\varepsilon Cl$, where A is the absorbance, ε the molar extinction coefficient (mol L^{-1} cm^{-1}), C the concentration of dyes or PhACs (mol L^{-1}) and l the path length (cm).

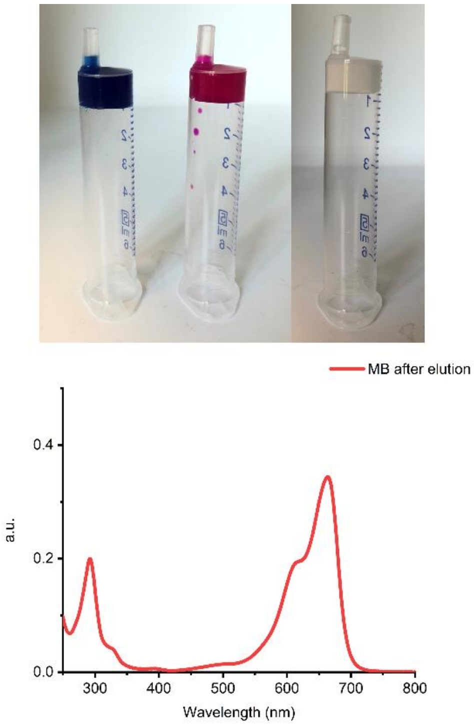

Figure S11: (Top) inversion of the syringe after the process to demonstrate the stability of the gel after the adsorption of pollutants; (bottom) UV-Vis spectrum of eluted MB to show the absence of Pyrene in the eluted sample.

Pollutant	Calibration curve slope (M^{-1})	Wavelength (nm)
Methylene Blue (MB)	23.325	663
Methyl Orange (MO) (pH=4.5)	11.221	485
Rhodamine B (RhB)	16.691	554
Diclofenac (DIC)	7.031	276
Ciprofloxacin (CIP)	5.0328	316
Nalidixic acid (NAX)	10.524	315
Carbamazepine (CBZ)	4.109	285

Table S1: Calibration curve slope and relative wavelength.

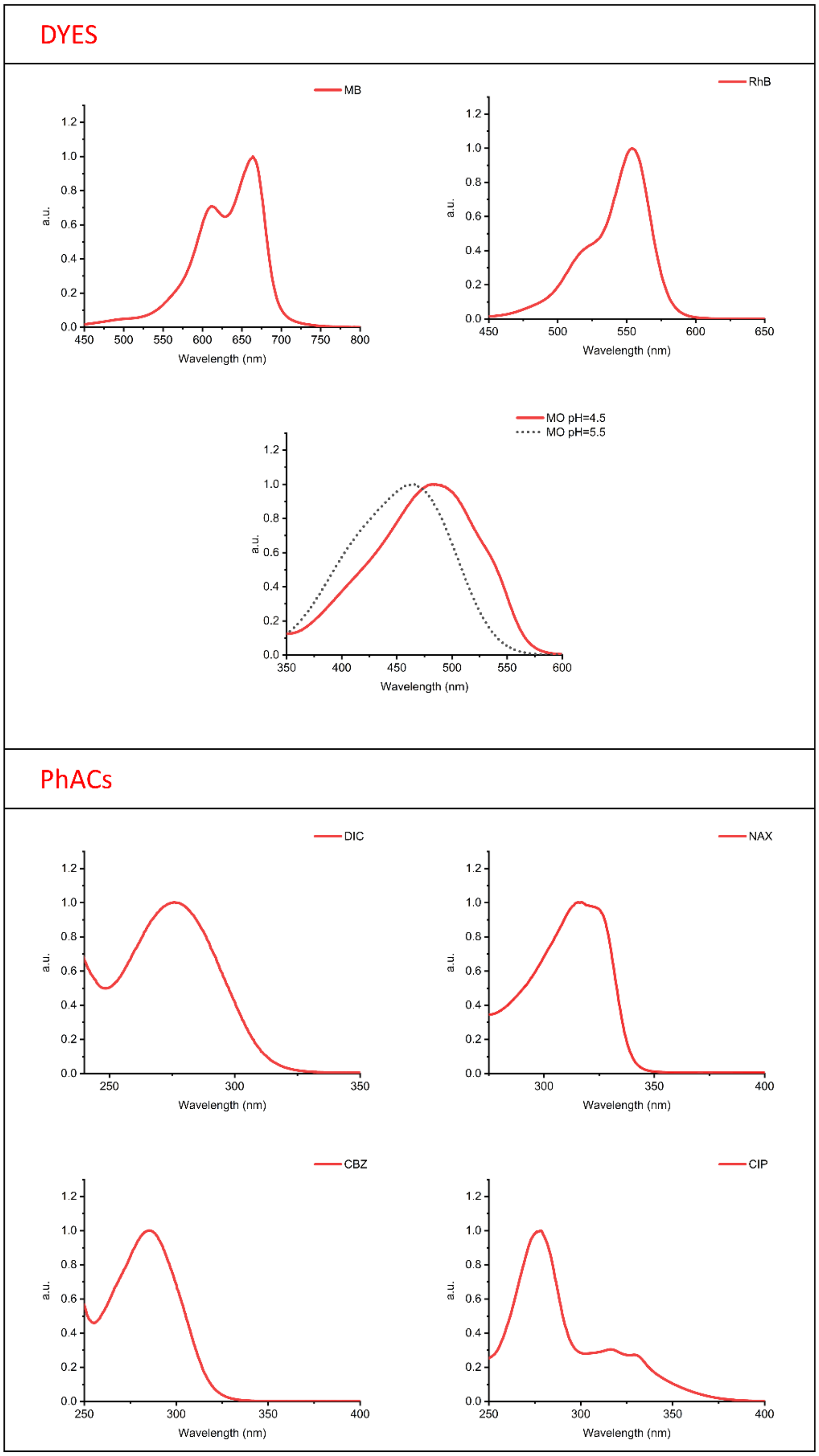

Figure S12: UV-VIs normalized absorption spectra of Dyes and PhACs used.

SYNTHESIS OF DEHALOGENATED DCF

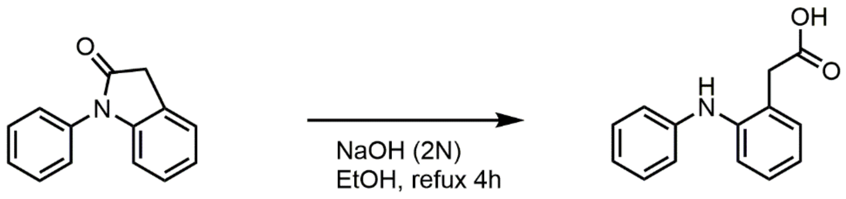

Scheme S2: Synthesis if dehalogenated DCF.

A solution of 500 mg of 1-Phenyloxindole in 2.4 ml of 2 N NaOH and 23ml of ethanol was refluxed for 4 hours. Ethanol was removed by evaporation. The solution was washed with diethyl ether. The residual mixture was then acidified using hydrochloric acid to a final pH=5. The precipitate obtained was then filtered, washed with water and air-dried.

ESI-MS: $[M+H]^+$, calculated for $C_{14}H_{13}NO_2$ 228.1, found: 228.2, $[M+Na]^+$, calculated for $C_{14}H_{13}NO_2$ 250.2, found: 250.1,.

^{1}H NMR (300 MHz, MeOD) δ 7.28-7.13 (m, 5H, Ar), 7.03-6.93 (m, 1H, NH), 6.91-6.72 (m, 3H, Ar), 3.63 (s, 2H, CH_2).

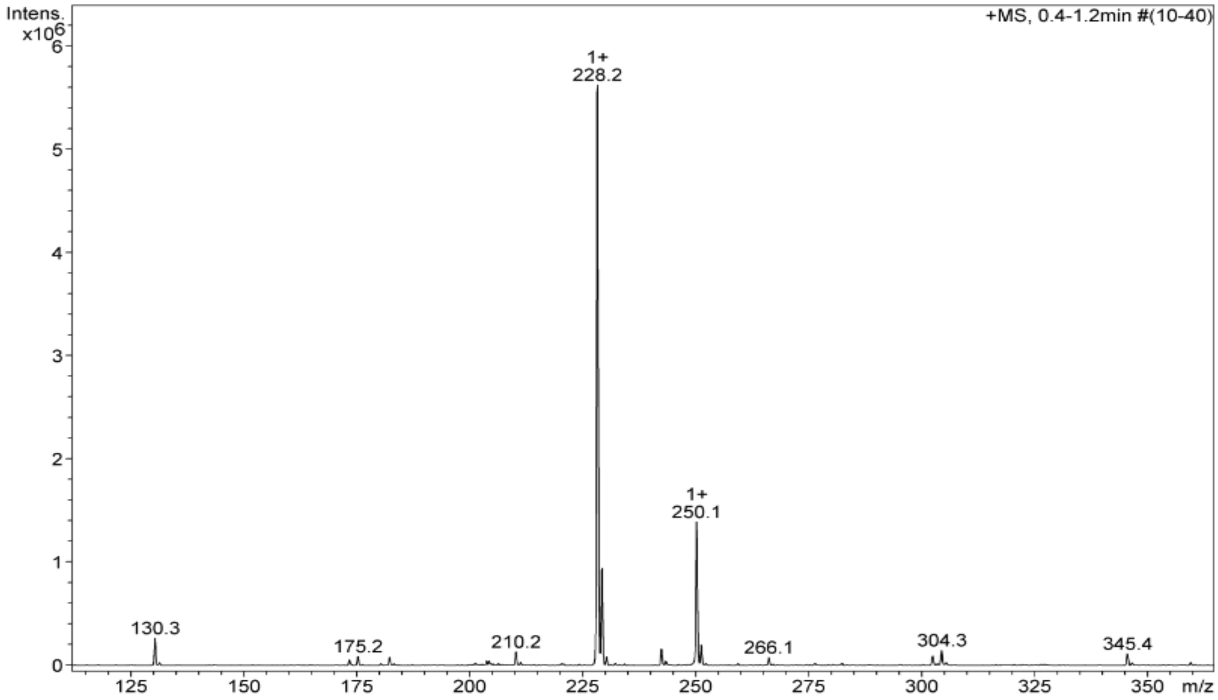

Figure S13: ESI-MS of 2-(2-(phenylamino)phenyl)acetic acid in MeOH.

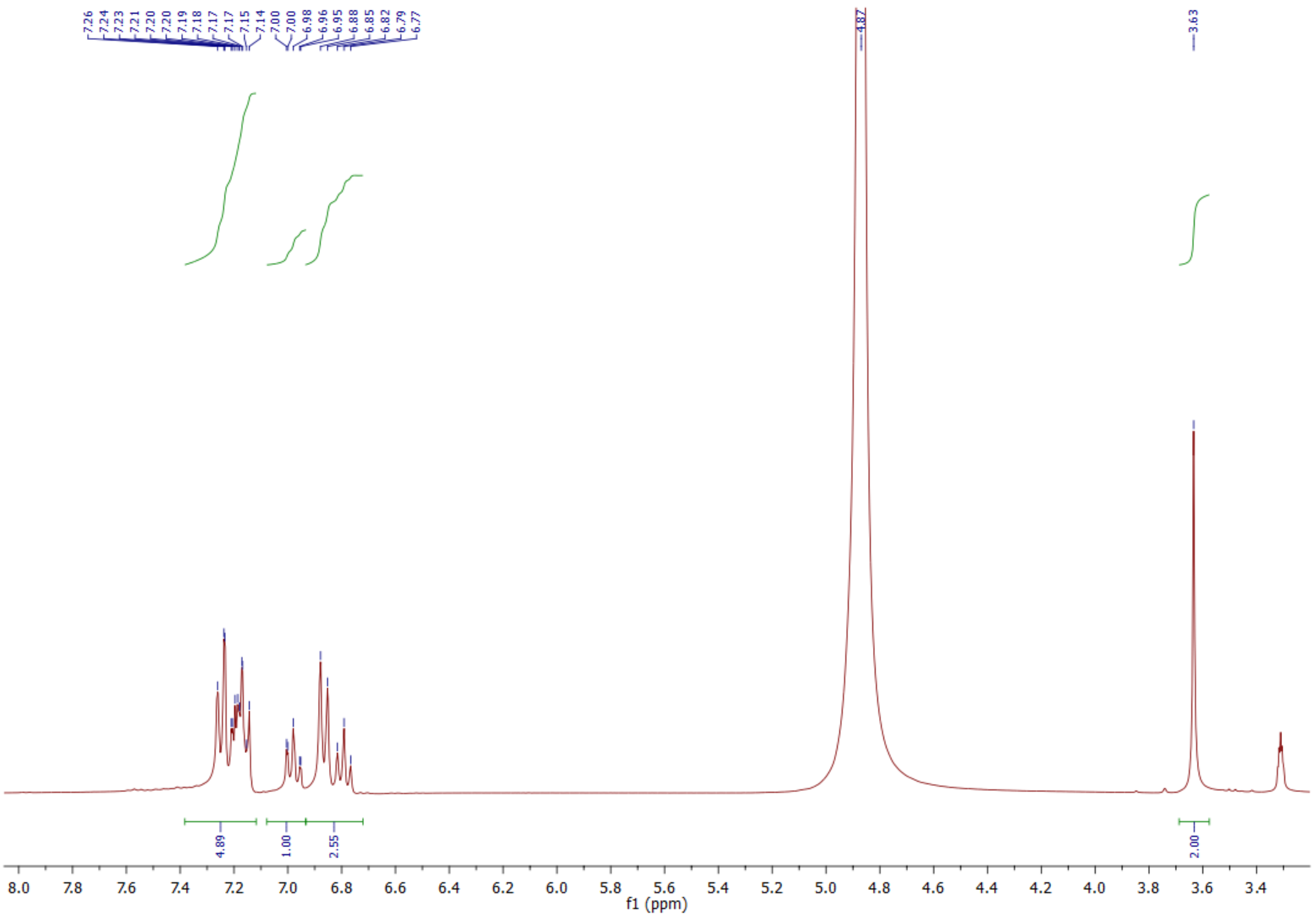

Figure S14: 1-NMR of dehalogenated DCF in MeOD.
